# Omnidirectional Structured Light in a Flexible Configuration

**DOI:** 10.3390/s131013903

**Published:** 2013-10-14

**Authors:** Carmen Paniagua, Luis Puig, José J. Guerrero

**Affiliations:** 1. Departamento de Informática e Ingeniería de Sistemas, Instituto de Investigación en Ingeniería de Aragón I3A, University of Zaragoza, E-50018, Spain; E-Mail: 578001@unizar.es; 2. GRASP Laboratory, University of Pennsylvania, 3330 Walnut Street, L402, Philadelphia, PA 19104, USA; E-Mail: luispuig@seas.upenn.edu

**Keywords:** structured light, omnidirectional camera, wearable system

## Abstract

Structured light is a perception method that allows us to obtain 3D information from images of the scene by projecting synthetic features with a light emitter. Traditionally, this method considers a rigid configuration, where the position and orientation of the light emitter with respect to the camera are known and calibrated beforehand. In this paper we propose a new omnidirectional structured light system in flexible configuration, which overcomes the rigidness of the traditional structured light systems. We propose the use of an omnidirectional camera combined with a conic pattern light, *i.e.,* the 3D information of the conic in the space. This reconstruction considers the recovery of the depth and orientation of the scene surface where the conic pattern is projected. One application of our proposed structured light system in flexible configuration consists of a wearable omnicamera with a low-cost laser in hand for visual impaired personal assistance.

## 1. Introduction

In computer vision, one of the most important goals is to obtain 3D information from the scene. This problem has been studied for many years [1]. In order to obtain this information there are two kind of methods: passive and active. The passive methods extract features from the scene such as corners, edges, lines, conics from textured images. The multiple observations of these features allow us to apply triangulation techniques [2] to recover the 3D information of the scene. One of the most known techniques where two aligned cameras are used to recover this information is stereo vision [3]. However, these techniques cannot be used when the texture of the scene contains few or no features. In these situations active methods are used. Among these systems, structured light [4] is the most popular. These systems are usually composed of a perspective camera and a light emitter which could either be a projector [5–7] or a laser projecting a known pattern [8–11]. The main goal of these systems is to obtain depth and surface orientation from the deformed patterns projected in the scene observed by the vision system. The most common configuration for these kind of systems is the rigid one, where the camera and the light emitter are fixed in a rigid configuration. Different calibration approaches have been developed for this type of systems [12–15], which computes the intrinsic parameters of the perspective camera and light emitter as well as the relative position with respect to one another. A recently presented approach locates the camera and the light emitter on a mobile platform with fixed position where the deformation of the projected pattern allows the computation of the platform position and orientation [16]. An evolution of these systems locate either the camera or the light emitter in a mobile platform, for example in a robotic arm [17,18]. Although this semi-rigid configuration provides more flexibility, the movements of the system are still limited and a calibration is required. The calibration process of these systems becomes more complex since it has to compute the relative position of the elements every time the moving part changes its position.

In this paper, we explore a new configuration for structured light systems, where both components, the camera and the light emitter are free to move in the space. We call this a non-rigid or flexible configuration. To our knowledge, a non-rigid configuration has only been used before in [19], where a scanning technique using a hand-held camera and a hand-held projector is presented. In our approach we use a wearable camera and a hand-held light emitter with a conic pattern. The conic pattern has a strong mathematical support and has been studied within the algebraic projective geometry [16,20,21]. We also propose the use of an omnidirectional camera where the light emitter is visible and from which its relative location is partially computable. Besides that, there are very few omnidirectional structured light systems but all of them in rigid configuration [22].

**Figure 1.**
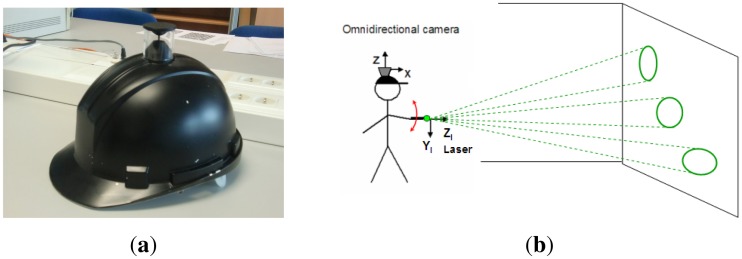
Flexible structured light system. (**a**) Wearable omnidirectional camera; (**b**) Configuration of the system.

Hence, in this work we present a novel omnidirectional structured light approach with a totally free motion of the conic pattern light emitter. In Figure 1 we show the wearable omnidirectional camera used [23] and the configuration of the system. We use the image of the light pattern acquired by the omnidirectional camera and a virtual image generated from the calibrated light emitter to perform the conic reconstruction algorithm. From this algorithm we compute the depth and orientation of the surface where the conic pattern has been projected. The ultimate goal of this system is the development of a wearable personal assistance system, with a low-cost laser in hand.

The remaining sections are organized as follows. In Section 2 the problem is formulated. We present the camera and laser models used as well as the conic correspondence condition. In Section 3 the 3D information of the projection plane is computed. In Section 4 several simulations and experiments are shown. Finally, conclusions and remarks are given in Section 5.

## 2. Problem Definition

In order to compute the depth of the scene we require to solve a conic reconstruction problem. In [24] two images in a rigid stereo configuration are proposed. Our approach captures one of such images from a calibrated omnidirectional camera. The second image is a virtual image obtained from the calibrated light emitter. Since the configuration we use is non-rigid the position and orientation of the laser is not available. In order to obtain such information we come up with the idea of using the omnidirectional image where the light emitter is always partially visible. In this section we present the camera and the laser model. We also present the conic correspondence condition, which is very useful to determine the correct position of the laser with respect to the camera.

### 2.1. Omnidirectional Camera Model

**Figure 2.**
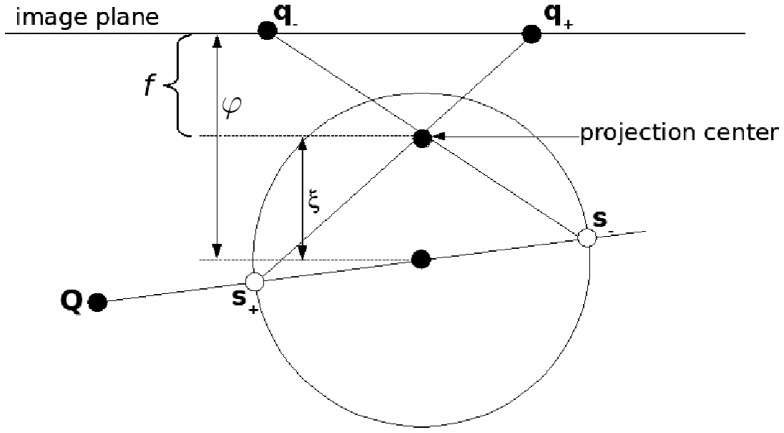
Projection of a 3D point to two image points in the sphere camera model.

We use the sphere model for catadioptric projection introduced by Geyer and Daniilidis in [25]. This model covers all central catadioptric cameras, encoded by *ξ*, which is the distance between the perspective camera and the center of the sphere, and *ψ,* which is the distance between the center of the sphere and the image plane. According to the model, the projection of 3D points **Q** can be performed in two steps (Figure 2). First, one projects the point onto the unit sphere, obtaining the intersection of the sphere and the line joining its center and the 3D point. There are two intersection points which are represented by **s**±. Second, these points are projected using a perspective projection P resulting in two image points, **q**±. Only one of these points is physically true. These steps are encoded in the non-linear function *ħ*:

(1) $$\hbar (\text{Q})={\left(Q_{1,}Q_{2,}Q_{3,}\pm \xi \sqrt{Q_{1}^{2}+Q_{2}^{2}+Q_{3}^{2}}\right)}^{\text{T}}$$

Its corresponding inverse function is *ħ*^−1^, which maps image points q into oriented 3D rays:

(2) $$\hbar^{-1}(\text{q})=\left(\begin{array}{c} \frac{q_{3}\xi +\sqrt{q_{3}^{2}+(1-\xi^{2})\left(q_{1}^{2}+q_{2}^{-2}\right)}}{q_{1}^{2}+q_{2}^{2}+q_{3}^{2}}q_{1}\\ \frac{q_{3}\xi +\sqrt{q_{3}^{2}+(1-\xi^{2})\left(q_{1}^{2}+q_{2}^{-2}\right)}}{q_{1}^{2}+q_{2}^{2}+q_{3}^{2}}q_{2}\\ \frac{q_{3}\xi +\sqrt{q_{3}^{2}+(1-\xi^{2})\left(q_{1}^{2}+q_{2}^{-2}\right)}}{q_{1}^{2}+q_{2}^{2}+q_{3}^{2}}q_{3}-\xi \end{array}\right)$$

### 2.2. Conic Laser Model

**Figure 3.**
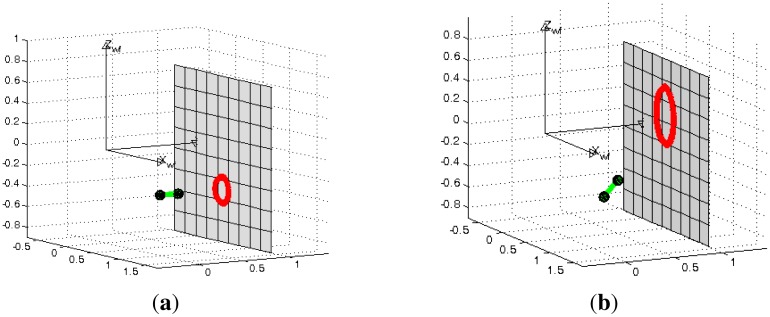
Laser model. (**a**) Calibration process; (**b**) Deformation of the conic shape depending on the laser orientation.

The projector model is the same as the pin-hole camera, since the projector can conceptually be regarded as an inverse camera, projecting rays on the scene. Its *z*-axis points in the direction of the laser projection. The projected pattern corresponds to a generic conic x^T^Cx = 0, where C is a symmetric 3 × 3 matrix and x are image points in projective coordinates [2]. If the radius of the projected pattern is known to a certain unitary distance we say the system is calibrated (see Figure 3a). If the orientation of the light emitter is also known, the shape of the projected conic is easily calculated (see Figure 3b). Since we consider the laser as an inverse pin-hole camera it is possible to create a virtual perspective image from the projected conic. This virtual image will be used subsequently to obtain the scene depth.

### 2.3. Conic Correspondence Condition

**Figure 4.**
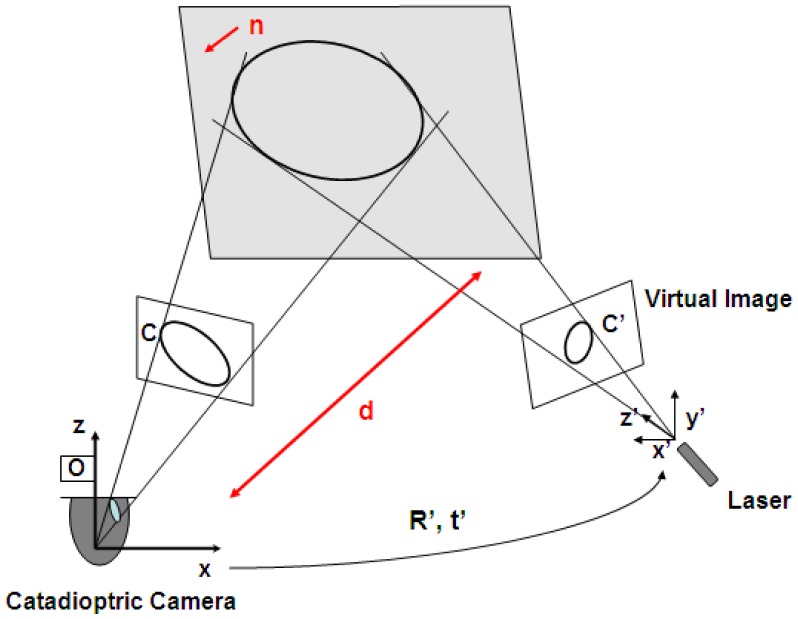
Depth information from conic correspondence using virtual images.

Two conics are corresponding when both of them are projections of the same conic in space (see Figure 4). For an image conic C we define its corresponding cone Q, which joins C and the projection center of the camera, defined by matrix P, as

(3) $$\begin{array}{ccc} {\text{x}}^{\text{T}}\text{Qx}=0, & \text{with} & \text{Q}={\text{P}}^{\text{T}}\text{CP} \end{array}$$

For a pair of corresponding conics C and C' we define their corresponding cones as

(4) Q≡xTPTCPx=xTAx;Q'≡xTP′TC′P′x=xTBx

from which we compute a pencil of quadric surfaces [24] as

(5) C(λ)=A+λB

with characteristic polynomial

(6) $$\left|\text{C}(\lambda )\right|=\left|\text{A}+\lambda \text{B}\right|=I_{1}\lambda^{4}+I_{2}\lambda^{3}+I_{3}\lambda^{2}+I_{4}\lambda +I_{5}$$

The coefficients *I_j_* are polynomials in the entries of A and B. These coefficients allow us to define λ as

(7) $$\lambda =-\frac{I_{3}}{2I_{2}}$$

and the so-called **conic correspondence condition**

(8) $$\Delta \equiv I_{3}^{2}-4I_{4}I_{2}=0$$

When this condition is satisfied, it guarantees that the two conics are projections of the same conic in the space. This conic correspondence condition has a relevant role in the proposed method to compute the orientation of the light emitter with respect to the camera, as will be shown in the following sections.

## 3. Depth Information Using a Structured Light System in Flexible Configuration

The recovery of 3D information from conic correspondences has been studied in [24,26]. In [26] Conomis proposes the use of homographies, but this approach requires at least two conics to be observed and matched in at least two images. On the other hand, Quan [24] presents a stereo configuration in which only one conic has to be observed in at least two images to compute the location of the projection plane in the scene. Our approach is based on the algorithm proposed by Quan where one image is given by the omnidirectional camera and the second is a virtual perspective image generated from the calibrated light emitter (see Figure 4).

### 3.1. Computing Laser 3D Location

The 3D location of the light emitter is required in order to generate the second image of the scene conic. We use a calibrated omnidirectional image where the light emitter is partially visible, helping to compute its relative 3D position and orientation with respect to the camera reference system.

#### 3.1.1. Laser Translation

From an omnidirectional image where the light emitter is observed, we can compute its translation with respect to the omnidirectional camera using the inverse sphere camera model given by Equation (2). This function allows us to obtain the translation up to scale. In order to recover the scale, we attach a small ball with known radius to one of the endpoints of the laser. It allows us to obtain the distance, *D*, between the catadioptric camera and the light emitter.

Let q be the image point associated to the ball center with its corresponding ray on the unitary sphere s = *ħ*^−1^(q). This ray indicates the direction from the image center to the laser. To calculate the distance *D* we use the expression (9) presented in [27] where r is the ball radius, $r_{im}^{-}$ is the minor semi-axis of the ellipse corresponding to the ball observation in the omnidirectional image, *ξ* and *φ* are mirror parameters and *f* the focal length, obtained from the calibration [28] and *R_im_* is the distance from the principal point to the center of the ball in the image.

(9) $$D=(\xi -\varphi )\frac{r}{r_{im}^{-}}\frac{1+\xi f\left(\xi ,\frac{R_{im}}{\left(\xi -\varphi \right)}\right)}{{\left(\xi +f\left(\xi ,\frac{R_{im}}{\left(\xi -\varphi \right)}\right)\right)}^{2}}$$

where

(10) $$f\left(\xi ,\frac{R_{im}}{\left(\xi -\varphi \right)}\right)=\frac{\sqrt{1+{\left(\frac{R_{im}}{\left(\xi -\varphi \right)}\right)}^{2}(1-\xi^{2})-\xi {\left(\frac{R_{im}}{\left(\xi -\varphi \right)}\right)}^{2}}}{1+{\left(\frac{R_{im}}{\left(\xi -\varphi \right)}\right)}^{2}}$$

#### 3.1.2. Laser Orientation

In this section we explain two alternate methods to compute the laser orientation with respect to the camera. They are based on the detection of either only one endpoint of the laser or their two endpoints.

**Figure 5.**
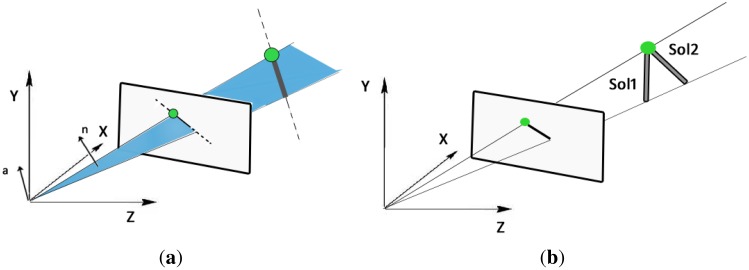
Methods to compute the orientation of the light emitter. (**a**) Method based on the plane defined by the laser and one endpoint; (**b**) Method based on the extraction of the laser endpoint and known length.

- **One-endpoint:** This method requires detecting one endpoint of the laser body and the center of the image conic to determine the projected orientation of the light emitter. Assuming a central projection system we can generate a projection plane using the projection center of the camera (see Figure 5a). Using the representation of projected lines and 3D lines proposed in [29], the normal vector of this projection plane, **n** = (*n_x_*, *n_y_*, *n_z_*), is used to calculate the rotation angles (*ϕ, θ*), which correspond to rotations around the *z*- and *y*-axis, respectively, defining the projection of the laser direction
  (11) $$\begin{array}{ll} \phi =\text{a}\tan2(n_{y},n_{x}) & \;\theta = \end{array}\text{a}\tan2\left(-n_{z},\sqrt{n_{x}^{2}+n_{y}^{2}}\right)$$
  These two angles (*ϕ*, *θ*) are obtained from the image. A third angle *ψ* around the *x*-axis, is required to compute the complete 3D orientation of the laser in such a way that its 3D orientation is
  (12) $$f(\phi ,\theta ,\psi )=\left(\begin{array}{c} \cos\phi \sin\theta \cos\psi +\sin\phi \sin\psi \\ \sin\phi \sin\theta \cos\psi -\cos\phi \sin\psi \\ \cos\theta \cos\psi \end{array}\right)$$
  and therefore the rotation from camera to the laser reference system is given by
  (13) $$\text{R}=rot_{z}(\phi )\cdot rot_{y}(\theta )\cdot rot_{x}(\psi )$$
  The computation of angle *ψ* is performed using a non-linear optimization method, in particular we use Matlab's Levenberg-Marquardt implementation, where the minimization criteria is the conic correspondence condition (8). We use as initial values *ψ* = 0 and *θ* and *φ* given by Equation (11).
- **Two-endpoints:** This method detects the whole body of the laser on the omnidirectional image and the sphere attached to it, from which we compute the corresponding 3D rays using Equation (2). With this information and the laser length there are only two possible solutions for the 3D orientation. This situation can be observed in Figure 5b. This ambiguity is solved as follows. For each solution we compute the corresponding projection cone and construct its pencil of quadric surfaces, from which we calculate the corresponding determinants. By evaluating (8) in the two solution we observe that only one satisfies the correspondence condition, being the correct orientation we are looking for. With the two endpoints correctly detected the computation of the 3D orientation is straightforward.

### 3.2. Projection Plane Location

As we mentioned before the scene information is given by the location of the projection plane in the scene. This information is obtained from the pencil of quadric surfaces C(λ) (see Equations (5) and (7)). In particular from its eigenvalues (*μ*_1_, *μ*_2_) and eigenvectors (**v**_1_, **v**_2_). There are two solutions for the plane where the conic lies. These planes are represented in Cartesian form **p***_i_* = (*A,B,C,D*), where (*A,B,C*) define the normal of the plane and *D* is the perpendicular distance from the camera to the plane.

(14) $$\begin{array}{l} {\text{p}}_{1}={\left(\sqrt{\mu_{1}}{\text{v}}_{1}+\sqrt{-\mu_{2}}{\text{v}}_{2}\right)}^{\text{T}}\text{x}=0\\ {\text{p}}_{0}={\left(\sqrt{\mu_{1}}{\text{v}}_{1}-\sqrt{-\mu_{2}}{\text{v}}_{2}\right)}^{\text{T}}\text{x}=0 \end{array}$$

To determine which one is the correct solution we use the projection centers of the two views o and o', given by the kernel (nullspace) of their corresponding projection matrices P and P'

(15) $$\begin{array}{lll} \text{o}=\text{Ker}(\text{P}) & \text{and} & {\text{o}}^{\prime }=\text{Ker}({\text{P}}^{\prime }) \end{array}$$

For non-transparent objects, the plane for which (o^T^p*_i_*)(o'^T^ p*_i_*), *i* = 1, 2 is positive represents the correct plane.

## 4. Experiments

To verify the validity of the proposed method, we perform experiments using simulated data and real images acquired with our omnidirectional structured light system with laser in hand. To measure the accuracy of the proposed approach we compute the distance from the camera to the projection plane and the orientation of the projection plane given by its normal.

### 4.1. Experiments with Simulated Data

**Figure 6.**
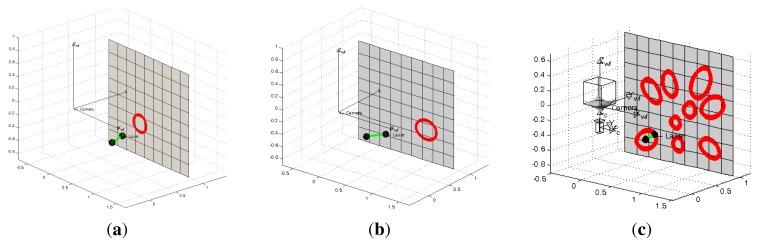
Different configurations of the proposed structured light system. (**a**) Single conic with azimuth 20°; (**b**) Single conic with azimuth 30°; (**c**) Multiple conics varying the azimuth.

In the first experiment we use the projection of a single conic in the scene. We tested different configurations of the system, varying the azimuth angles of the light emitter from −35° to 35° in intervals of five degrees with a constant elevation of zero degrees. The projection plane is located at one meter from the camera. We also add errors in the range [−5°, 5°] every degree to the angles *ϕ* and *θ* for the One-endpoint method and to the angles *ϕ, θ* and *ψ* for the Two-endpoint method. In Figure 6 we observe examples of these configurations.

**Figure 7.**
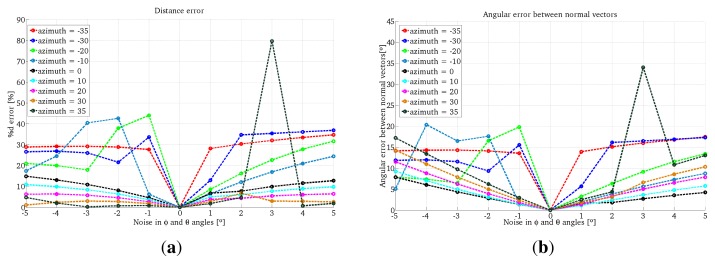
Simulation results using One-endpoint method with one conic projection. (**a**) Error in depth; (**b**) Error in the surface orientation.

**Figure 8.**
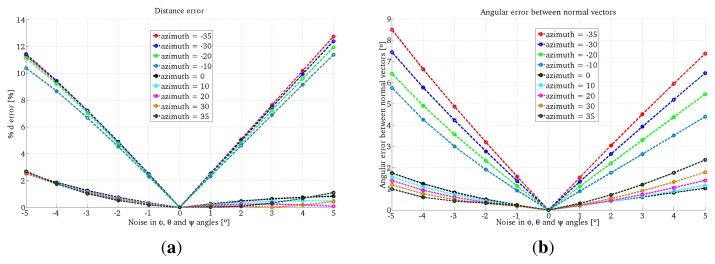
Simulation results using Two-endpoint method with one conic projection. (**a**) Error in depth; (**b**) Error in the surface orientation.

The results for the One-endpoint method and the Two-endpoint method are shown in Figures 7 and 8, respectively. We observe that the errors given by the One-endpoint method are higher than the ones given by the Two-endpoints method. This is explained because the light emitter location given by the Two-endpoints method has less than the One-endpoint method. We also observe that for some configurations the error in both methods increases considerably.

**Figure 9.**
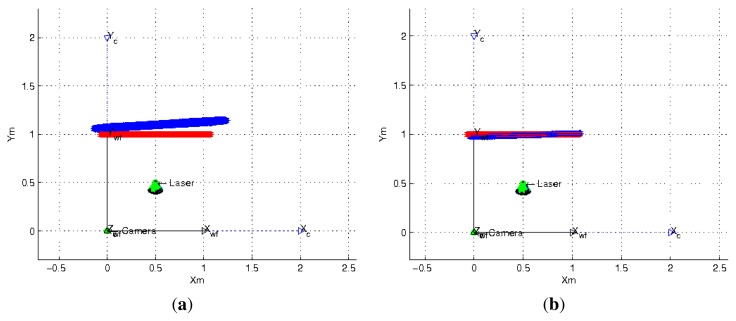
Estimation of a plane located 1*m* from the camera. Real plane in red and estimated plane in blue. (**a**) Plane computed using One-endpoint method; (**b**) Plane computed using Two-endpoint method.

In the proposed flexible configuration of the structured light system it is possible to acquire multiple observations of the same projection plane by moving the laser in hand while the omnidirectional camera is static. In Figure 6c we observe an example of this situation. We exploited this situation in the following experiment by combining all the nine observations from the last experiment where 5° noise is added to the three angles which represent the light emitter orientation and its projection in the image. Since there are incorrect estimations, we use a RANSAC algorithm adapted to planes estimation to avoid outliers. We use two different error criteria, the difference between the distances from the planes to the camera and the angle between their normal vectors. The results of this experiment are shown in Table 1. We observe that the estimation of the plane information improves considerably and that the Two-endpoint method obtains a better estimation than the One-endpoint method. In Figure 9 we observe the top-view of the plane estimation with both methods of laser orientation estimation.

### 4.2. Experiment Using a Real Omnidirectional Structured Light System in Flexible Configuration

**Figure 10.**
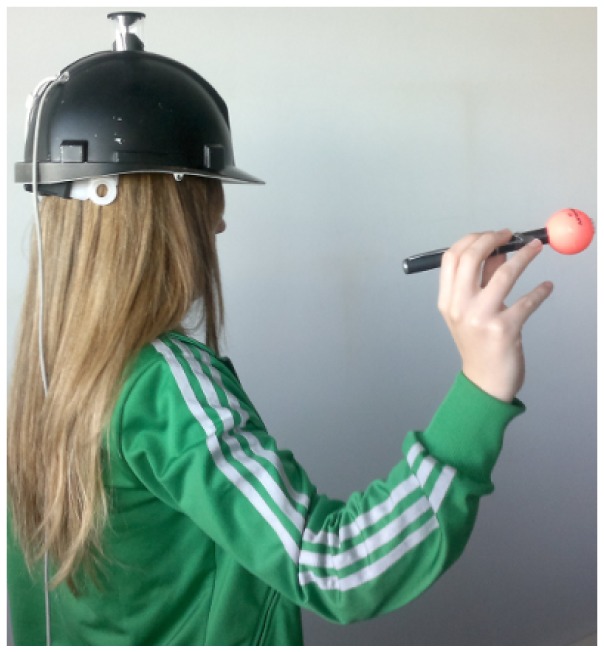
Wearable flexible structured light system with light emitter in hand.

These experiments are performed using our wearable flexible structured light system, which is composed of a catadioptric camera designed by Vstone [31], mounted on a helmet (see Figure 10) and a low-cost laser in hand projecting a conic pattern. The distance between the real plane and the camera is measured using the laser meter Leica DISTO D5, with an accuracy of ±1 mm on a rank of 200 m, in order to have a ground truth to evaluate the results.

As explained before our method to estimate the plane projection depth is based in acquiring multiple observations of the conic pattern projected on this plane while omnidirectional camera is static. In these experiments we use seven omnidirectional images of the projected conic pattern. Illumination conditions are adequate to extract at the same time the conic pattern and the light emitter with the sphere attached to it. We perform two experiments, one for each orientation method of the light emitter. In the first experiment the plane is located at 1.3 m and in the second one the plane is located at 1 m.

#### 4.2.1. Image Processing

**Figure 11.**
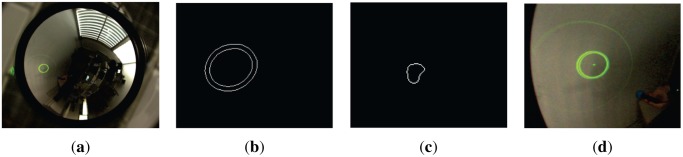
(**a**) Original image; (**b**) Pattern light extracted; (**c**) Laser endpoint extracted; (**d**) Light pattern detection.

We use the HSI (Hue Saturation Intensity) space color since it is compatible with the vision psychology of human eyes [30] and the independence of its three components. The process to extract the conic pattern and the spheres is the following. Using different thresholds in channels H and S we binarize the image, then edges are detected using the Canny algorithm. The connected pixels are stored in components. Using a five-point RANSAC approach for connected components we extract and estimate the conics present in the image. One for the laser ball and other for the projected light pattern. These steps are briefly shown in Figure 11.

Once the light pattern and the light emitter endpoints are extracted from the omnidirectional image we apply our method to obtain the depth information of the scene. We use the seven obtained solutions to compute a final one with a RANSAC algorithm adapted to planes as we have explained previously. The results of these experiments using the two orientation methods are shown in Table 2. These results validate the feasibility of our approach in real scenarios. The estimation of the orientation of the light emitter is better when we extract its two endpoints.

We observe that this step along with the non-linear optimization step are the most time consuming tasks. In this paper we use Matlab but a proper implementation in C/C++, like the OpenCV library, would improve the efficiency of our approach.

## 5. Conclusions

In this paper, we present a new omnidirectional structured light system in flexible configuration which can be used as a personal assistance system. We use it to recover the scene structure. This system only requires a single omnidirectional image where the light pattern and the light emitter are present. From this image the position and orientation of the laser in the space are computed. Up to our knowledge this is the first structured light system in flexible configuration. Our approach has shown good results for simulated and real data. The orientation of the light emitter is better estimated when its two endpoints are visible, having a big impact on the computation of the depth scene. In future work, we expect to improve the image processing step in order to deal with more general illumination conditions.

## Figures and Tables

**Table 1.** Scene depth information using simulated data and multiple azimuth observations of the conic pattern. A noise of 5° is added to the three angles representing the laser orientation and its projection in the image.

	**Distance (*d*)/Error (%)**	**Normal (n)/Error** (°)
Ground Truth	1m	(0,1,0)
One-endpoint method	1.03 m/2.87%	(−0.061, 0.995, 0.078)/5.74°
Two-endpoint method	0.97 m/2.78%	(−0.0294, 0.999, 0.009)/1.77°

**Table 2.** Distance estimated using our flexible structured light system.

	**One-Endpoint Method**	**Two-Endpoints Method**
Ground Truth	1.3 m	1.3 m
Estimated distance	1.17 m	1.26 m
Error	9.58%	3.14%
